# A Flexible Hot-Film Sensor Array for Underwater Shear Stress and Transition Measurement

**DOI:** 10.3390/s18103469

**Published:** 2018-10-15

**Authors:** Baoyun Sun, Pengbin Wang, Jian Luo, Jinjun Deng, Shiqi Guo, Binghe Ma

**Affiliations:** 1 Key Laboratory of Micro/Nano Systems for Aerospace, Ministry of Education, Northwestern Polytechnical University, Xi’an 710072, Shaanxi, China; sunbaoyun@mail.nwpu.edu.cn (B.S.); wangpb@mail.nwpu.edu.cn (P.W.); jian.luo@nwpu.edu.cn (J.L.); dengjj@nwpu.edu.cn (J.D.); 2 Department of Electrical and Computer Engineering, George Washington University, Washington, DC 20052, USA; gsq029@gwu.edu

**Keywords:** flexible, hot-film sensor, shear stress, transition, underwater

## Abstract

A flexible hot-film sensor array for wall shear stress, flow separation, and transition measurement has been fabricated and implemented in experiments. Parylene C waterproof layer is vapor phase deposited to encapsulate the sensor. Experimental studies of shear stress and flow transition on a flat plate have been undertaken in a water tunnel with the sensor array. Compared with the shear stress derived from velocity profile and empirical formulas, the measuring errors of the hot-film sensors are less than 5%. In addition, boundary layer transition of the flat plate has also been detected successfully. Ensemble-averaged mean, normalized root mean square, and power spectra of the sensor output voltage indicate that the Reynolds number when transition begins at where the sensor array located is 1.82 × 10^5^, 50% intermittency transition is 2.52 × 10^5^, and transition finishes is 3.96 × 10^5^. These results have a good agreement with the transition Reynolds numbers, as measured by the Laser Doppler Velocimetry (LDV) system.

## 1. Introduction

Wall shear stress is one of the most important fluid mechanical parameters in the boundary layer, which is a tangential force in the direction of motion due to the viscosity of the fluid [1,2]. The time averaged value of shear stress indicates the local shin friction drag, its measurement is essential for aerodynamic performance optimization of aircraft and drag reduction of underwater vehicle. The time resolved value of shear stress can be used to analyze the flow fluctuation, like vortex structure, coherent portion of turbulence, and so on [3]. Thus, the accurate and time-resolved measurement of shear stress is critical for a physical understanding of complex flow phenomena and assisting in their control [4].

Transition from laminar to turbulent flow drastically changes the skin friction drag, aerodynamic/hydrodynamic noise, vortex structure, and heat transfer rate of fluids, yet when and how turbulence emerges is complicated even for simple flow. Due to its fundamental importance to the research of fluid motions, the transition phenomenon has received and continues to attract much attention. A great deal of theoretical and experimental work has been done on the instability of laminar boundary layers and in determining criteria for transition [5,6,7,8]. Because there are so many factors affecting transition, no satisfactory theory for the transition process in any flow condition has been established so far. Hence, the ability to experimentally determine transition Reynolds number and location is very important for turbulence studies.

Over the last few decades, various methods have been developed for shear stress measurement [9,10,11], such as Preston or Stanton tubes, laser Doppler anemometers, sublayer fence, floating element sensors, and hot-wire or hot-film probes, etc. Among all of these approaches, the flexible hot-film sensor fabricate on polymer substrate with a waterproof cover is most suitable for underwater shear stress and transition measurements, since it has good water resistance, high temporal/spatial resolution, less interference to the flow, and is well fit on curved surfaces.

Many qualitative flow separation and transition detection with hot-film sensors have been conducted in the air [12,13,14,15]. However, these flow phenomena measurement experiments can hardly be seen in the water, especially quantitative shear stress measurement and flow transition detection. In this paper, a flexible hot-film sensor array with deposited Parylene C as the waterproof cover layer is developed for underwater flow measurement. The quantitative shear stress and transition measurement experiments on a flat plate were conducted in a water tunnel. The results have a good agreement with the empirical formulas and Laser Doppler Velocimetry (LDV) velocity profile.

## 2. Flexible Hot-Film Sensor

### 2.1. Working Principle

**Figure 1:**
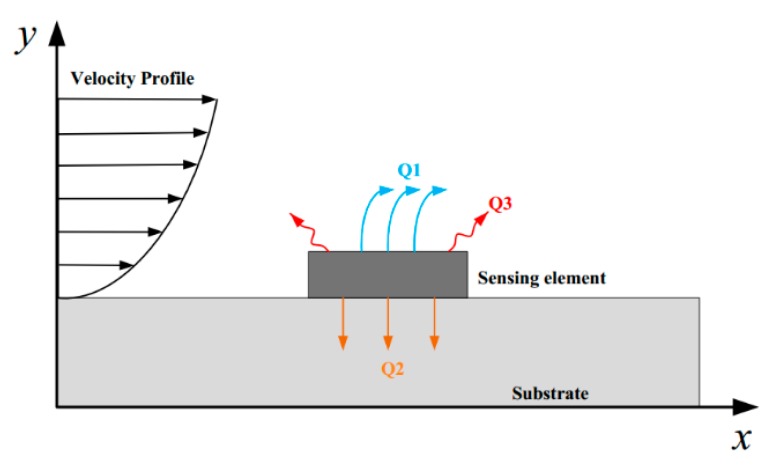
Schematic view of the hot-film sensor working principle.

Basically, a hot-film sensor makes use of the principle of heat transfer from a thermistor heated by current to the passing flow [16]. As shown in Figure 1, the heat generated by the thermistor dissipated into the ambient in three ways, heat convection to the flow (Q_1_), heat conduction to the substrate (Q_2_), and heat radiation (Q_3_), while the heat radiation can be neglected. The rate of heat loss from the heated thermistor to the flow is dependent on the velocity profile in the boundary layer. The wall shear stress *τ*_w_ generated by the flow is expressed as [1],

(1) $$\tau_{w}=\mu \frac{\partial U}{\partial y},$$

where *μ* is the fluid’s viscosity, *∂*U/*∂*y is the mean streamwise velocity gradient at the wall. As the temperature of the thermistor varies with changes of the velocity gradient in the boundary layer, so does the resistance. Thus, we can obtain the shear stress by means of the sensor output voltage indirectly.

The relationship between the shear stress *τ* and input power P to the sensor is typically described by the following empirical equation [17,18],

(2) $$P=\frac{U^{2}}{R}=\Delta T\left[A_{t}{\left(\rho \tau \right)}^{\frac{1}{m}}+B_{t}\right],$$

where U and R are the output voltage and the resistance of the shear stress sensor at the operating temperature T, respectively. ∆T is the temperature difference between the sensor operating temperature T and fluid T_f_, *ρ* is the density of the fluid, A_t_, B_t_, and m are usually determined by calibration. When the sensor working in constant current (CC) mode, the input current I is constant. According to ohm’s law, U = IR. Equation (2) can be expressed as,

(3) $$\mathrm{UI}=\left(T-T_{f}\right)\left[K_{1}\tau^{n}+K_{2}\right],$$

where K_1_ = A_t_*ρ*^1/m^, K_2_ = B_t_, n = 1/m. When the shear stress is zero, we have

(4) $$U_{0}I=\left(T_{0}-T_{f}\right)K_{2}.$$

where T_0_ is the operating temperature when the shear stress is zero, U_0_ is the output voltage at the operating temperature T_0_. Hence, the temperature difference between T_0_ and T can be derived from Equations (3) and (4),

(5) $$T_{0}-T=I\left(\frac{U_{0}}{K_{2}}-\frac{U}{K_{1}\tau^{n}+K_{2}}\right).$$

The temperature coefficient of resistance (TCR) α is an important parameter for the operation of the thermal shear stress sensor, which is given by,

(6) $$\alpha =\frac{{R-R}_{\mathrm{ref}}}{(T-T_{ref})R_{ref}},$$

where R is the resistance at temperature T, R_ref_ is the reference resistance at reference temperature T_ref_. So, the resistance R at operating temperature T and the resistance R_0_ at operating temperature T_0_ can be described by the following Equations (7) and (8), respectively.

(7) $${R=R}_{ref}\left[1+\alpha \left(T-T_{ref}\right)\right],$$

(8) $$R_{0}{=R}_{ref}\left[1+\alpha \left(T_{0}-T_{ref}\right)\right].$$

The temperature difference between T_0_ and T can also be derived from Equations (7) and (8),

(9) $$T_{0}-T=\frac{R_{0}-R}{R_{ref}\alpha }=\frac{{I(R}_{0}-R)}{{\mathrm{IR}}_{ref}\alpha }=\frac{U_{0}-U}{{\mathrm{IR}}_{ref}\alpha }.$$

Finally, the relationship between U and U_0_ can be derived from Equations (5) and (9),

(10) $${U=U}_{0}-\frac{A\tau^{n}}{1+B\tau^{n}},$$

where $A=\frac{U_{0}K_{1}}{K_{2}(I^{2}R_{\mathrm{ref}}\alpha K_{2}-1)}$, $B=\frac{I^{2}R_{\mathrm{ref}}\alpha K_{1}}{I^{2}R_{\mathrm{ref}}\alpha K_{2}-1}$.

### 2.2. Fabrication

**Figure 2:**
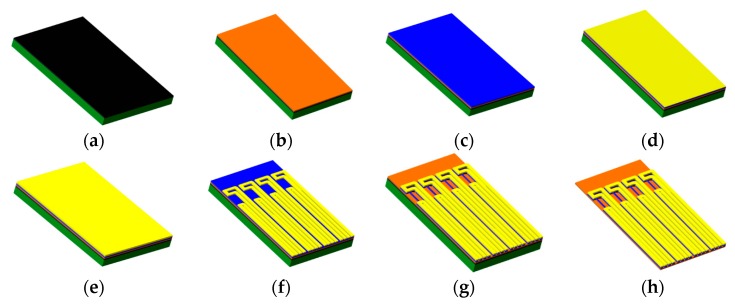
Fabrication processes of the flexible hot-film sensor array.

**Figure 3:**
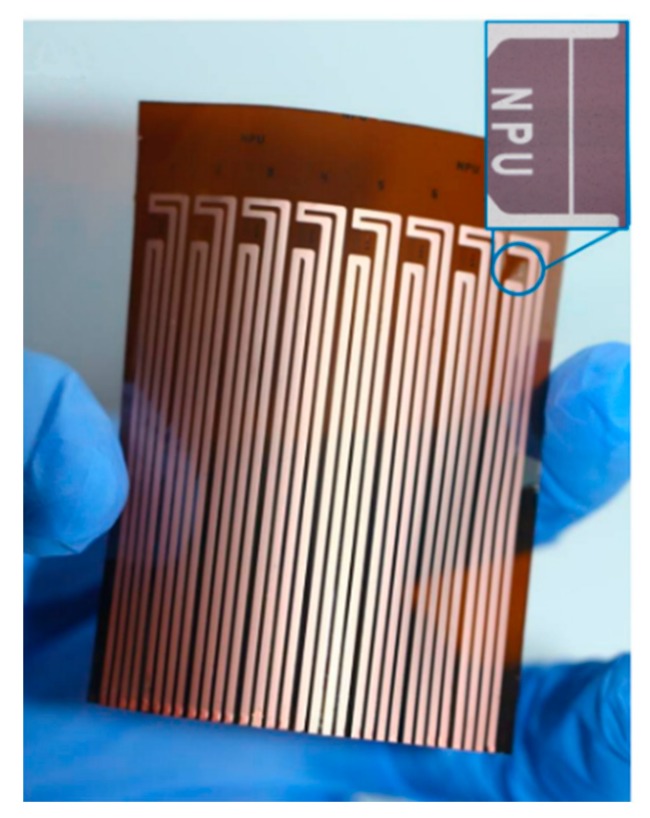
Flexible hot-film sensor array with eight sensors.

The fabrication processes of the flexible hot-film sensor array are shown in Figure 2 [19]. A ready-made polyimide (PI) foil in the thickness of 50 μm is used as the sensor substrate. It is affixed onto a glass wafer by using a polydimethylsiloxane (PDMS) adhesive layer (b), which is spun-on the wafer. Thermal sensing nickel (Ni) layer of 1 μm is then magnetron sputtered (c) onto the cleaned PI foil. With a 200 nm thick sputtered copper seed layer (d), the copper tracks layer is electroplated (e) to 2 μm. Then a first photolithography step is carried out and the tracks are wet-etched (f). Afterwards, the sensing elements are patterned with a second photolithography and wet-etched processes (g). The sensing element is 3 mm long and 50 μm wide. Finally, the sensor array is released from the glass (h). The fabricated sensor array with eight sensors is shown in Figure 3.

The sensor sensitivity and dynamic characteristic is proportional to the TCR [20]. Hence, to reach a better sensor performance, the value of TCR must be as high as possible. Vacuum thermal annealing is carried out to improve the TCR value and to eliminate lattice defects in Ni film. The sensor is kept in a high-vacuum furnace, the temperature increased to 400 °C within 120 min and kept constant for 360 min.

To operate under water, a waterproof layer needs to be deposited to encapsulate the sensor. Parylene C is chosen as the waterproof material due to its small moisture transmission [21]. 2 μm thick Parylene C waterproof layer is vapor phase deposited. Underwater shear stress and transition measurements that are introduced in the following chapters validate the reliability of Parylene C as a waterproof layer.

### 2.3. Calibration

**Figure 4:**
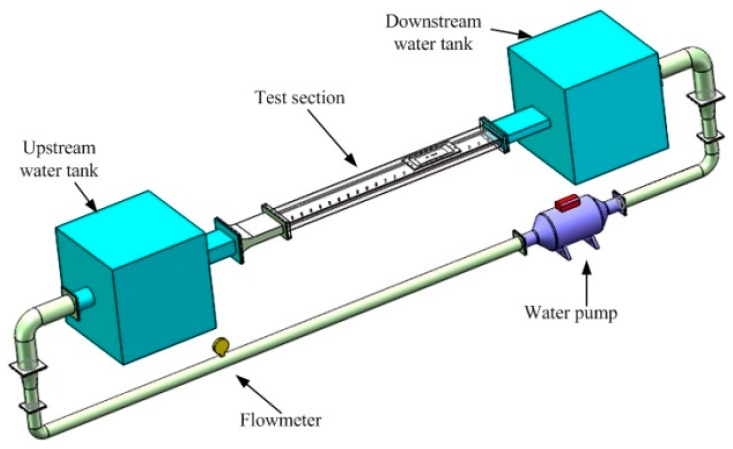
Schematic diagram of the water tunnel.

**Figure 5:**
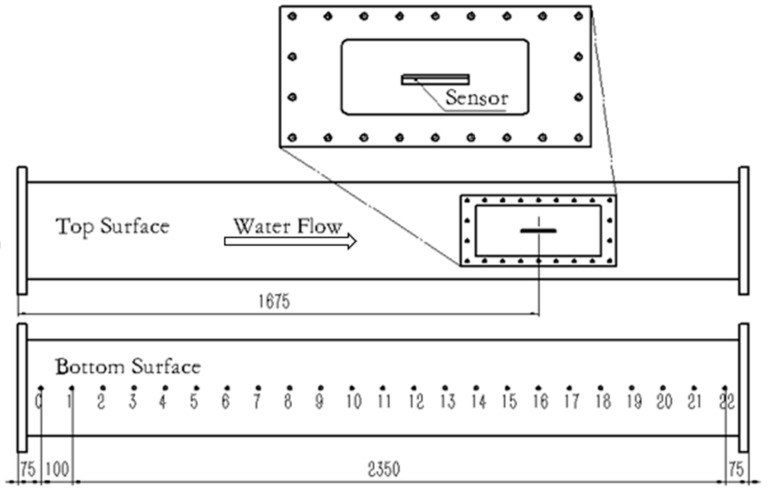
Schematic drawing of the test section.

The sensor array calibration was carried out in a water tunnel. The schematic drawing of the water tunnel facility is shown in Figure 4, it mainly consists of upstream and downstream water tanks, water pump, electromagnetic flowmeter, and test section. The test section channel has a length of 2600 mm, a width of 250 mm, and a height of 20 mm. The wall shear-stress in the channel is obtained from the measurement of the pressure gradient along its length. As illustrated in Figure 5, there are 23 pressure taps that are distributed uniformly at the lower wall of the test section. The first tap is 75 mm from the inlet of the test section, and the distance between every two neighboring taps is 100 mm. The shear stress can be calculated by the following equation,

(11) $$\tau_{w}=\frac{h}{2}\frac{dp}{dx},$$

where h is the height of the channel, dp/dx is the differential pressure drop. We have conducted CFD simulations and LDV measurements about the shear stress and flow development in the test section before. The shear stress measured by pressure gradient have a good agreement with the numerical simulation and LDV results [22,23].

**Figure 6:**
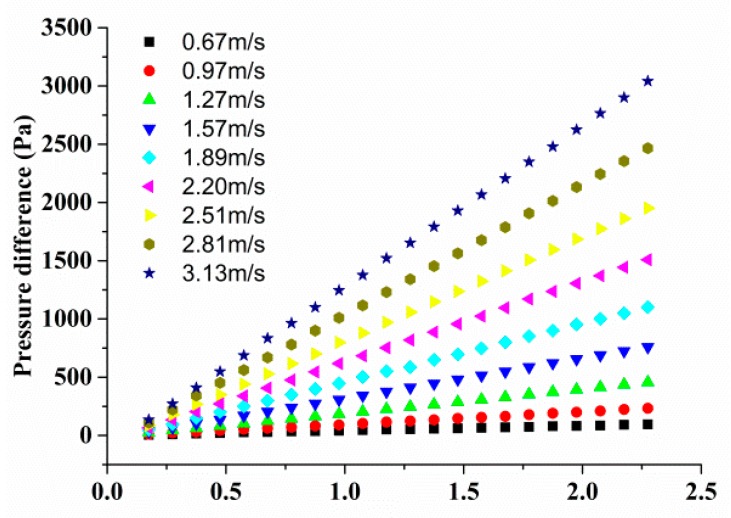
Pressure difference with various velocities.

In the calibration experimental setup, the sensors were surface-flush mounted on the center line of the upper wall of the test section (Figure 5). The eight sensors in the array were located from 1654 mm (sensor 1) to 1696 mm (sensor 8) from the inlet of the test section, and the space between two neighboring sensors is 6mm. These distances from the inlet of the test section to the sensors are sufficient to ensure that the flow is fully developed and effectively two-dimensional [22,23]. The hot-film sensors were working under CC mode, and the driving current were 50 mA. The steady-state sensor output voltages were measured by a data acquisition system (National Instruments) with a sampling frequency of 12 kHz for 8 s. The water temperature was 16 °C and unchanged during the calibration. The pressure difference with various velocities is shown in Figure 6.

**Figure 7:**
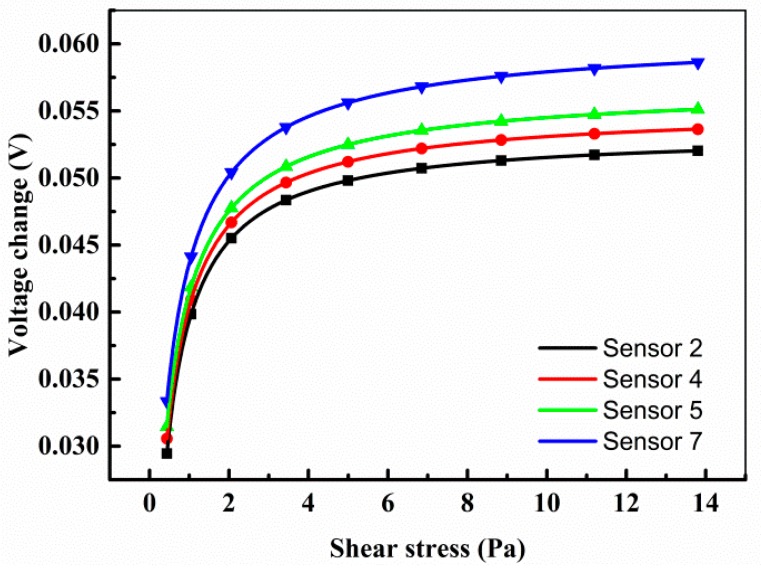
Calibration curves of four sensors.

The calibration curves of sensor 2, sensor 4, sensor 5, and sensor 7 are shown in Figure 7, respectively. According to Equation (10), the relationship between voltage change ΔU (V) and shear stress *τ*_w_ (Pa) can be expressed by the following equations,

(12) $$Sensor\;2:\Delta U=\frac{0.1498\tau_{w}^{0.9963}}{1+2.807\tau_{w}^{0.9963}},$$

(13) $$Sensor\;4:\Delta U=\frac{0.15\tau_{w}^{0.956}}{1+2.733\tau_{w}^{0.956}},$$

(14) $$Sensor\;5:\Delta U=\frac{0.151\tau_{w}^{0.928}}{1+2.662\tau_{w}^{0.928}},$$

(15) $$Sensor\;7:\Delta U=\frac{0.1542\tau_{w}^{0.8912}}{1+2.534\tau_{w}^{0.8912}},$$

**Figure 8:**
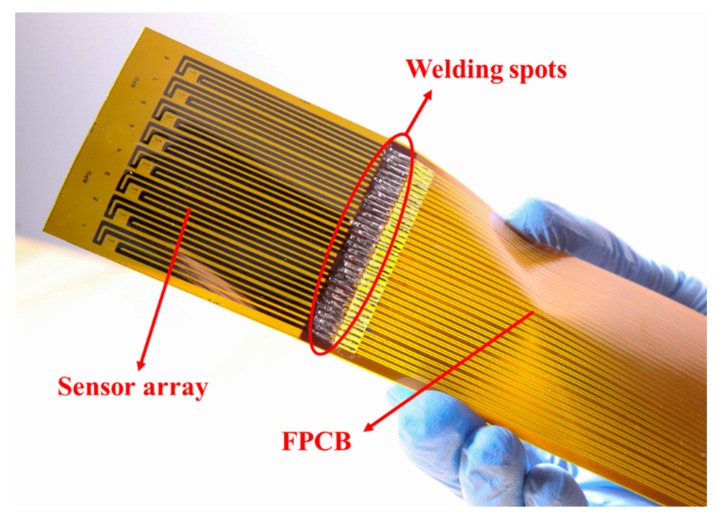
Sensors are connected to the flexible printed circuit board (FPCB) by welding.

The other four sensors have no output signal after we peeled off the sensor array from the test section. After test, the sensors were all still functional, but the welding spots (Figure 8) connecting the sensors and the flexible printed circuit board (FPCB) were broken. These damages were very random and occasional. If we did these works more carefully, then these damages can be avoided. However, the robustness of the welding spots really needs to be improved.

## 3. Shear Stress Measurement

**Figure 9:**
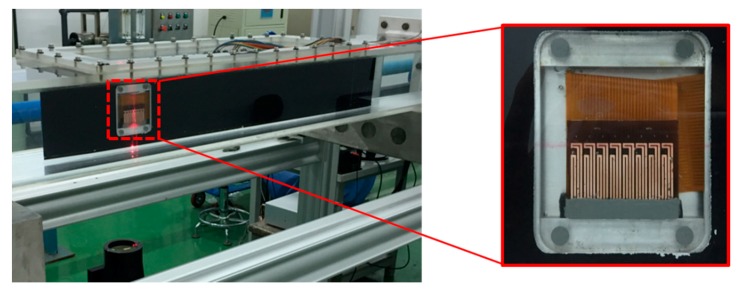
Flexible hot-film sensor array mounted on the flat plate in the water tunnel.

Underwater shear stress measurement experiments were conducted in the same water tunnel facility, as illustrated in Figure 4, but the test section has been replaced by a new one. The length of the new test section is 2600 mm, width is 250 mm, and height is 150 mm. A photograph of the flat plate installed in the center of the test section and the hot-film sensor array flush mounted on the flat plate surface are shown in Figure 9. The flat plate has a length of 1000 mm, a width of 150 mm, and a thickness of 20 mm. The angle between the flat plate and the center line of the test section (or the free-stream direction) is 0.34°, which can ensure zero pressure gradient on the flat plate. This angle was determined by pressure measurements. The sensors that were used for shear stress measurement were the calibrated ones in Section 2.3. The sensors were located from x = 780 mm (sensor 1) to x = 822 mm (sensor 8) from the leading edge of the flat plate. Except the flexible hot-film sensors, the LDV system and empirical formulas were also used to measure and calculate the shear stress of the flat plate.

### 3.1. Shear Stress Measurement by Hot-Film Sensors

The hot-film sensors were working under CC mode, and the driving current were 50 mA. The sensor output voltages with respect to various velocities were measured by a data acquisition system with a sampling frequency of 12 kHz for 8 s. The water temperature was kept at a constant of 16 °C. The velocity gradients at where the sensors mounted were measured by the LDV system. According to Equations (13)–(15), the shear stress *τ*_w_ (Pa) can be obtained by the voltage change ΔU (V),

(16) $$Sensor\;4:\tau_{w}=(\frac{\Delta U}{0.15-2.733\Delta U}{)}^{\frac{1}{0.956}},$$

(17) $$Sensor\;5:\tau_{w}=(\frac{\Delta U}{0.151-2.662\Delta U}{)}^{\frac{1}{0.928}},$$

(18) $$Sensor\;7:\tau_{w}=(\frac{\Delta U}{0.154-2.534\Delta U}{)}^{\frac{1}{0.891}},$$

the shear stress measured by sensor 4, sensor 5 and sensor 7 are shown in Table 1. One lead wire of sensor 2 was damaged during the test, so the data of sensor 2 was invalid.

### 3.2. Shear Stress Calculated by Empirical Formula

As known, the three full equations of motion can be reduced to Prandtl’s two boundary layer equations for two-dimensional incompressible flow [24],

(19) $$\frac{\partial u}{\partial x}+\frac{\partial \nu }{\partial y}=0,$$

(20) $$\mu \frac{\partial u}{\partial x}+\nu \frac{\partial \nu }{\partial y}=U\frac{dU}{dx}+\frac{1}{\rho }\frac{\partial \tau }{\partial y}.$$

There are two boundary conditions on u and one on ν: At y = 0, u = *ν* = 0; As y = *δ*(x), u = U(x). For laminar flow past the plate, the boundary layer equations can be solved exactly for u and *ν*, assuming that the free-stream velocity U is constant (dU/dx = 0). The solution was by the Blasius equation. The boundary thickness *δ* and wall shear stress *τ*_w_ can be governed by the following equations,

(21) $$\delta =\frac{5.0x}{Re_{x}^{1/2}},$$

(22) $$\tau_{w}=\frac{0.332\rho U^{2}}{Re_{x}^{1/2}}.$$

As there is no exact theory for turbulent boundary layer, two rough empirical formulas we used to calculated turbulent boundary layer thickness and shear stress are as follows [24],

(23) $$\delta =\frac{0.16x}{Re_{x}^{1/7}},$$

(24) $$\tau_{w}=\frac{0.0135\rho U^{2}}{Re_{x}^{1/7}}.$$

The boundary layer thickness at where sensor 5 located calculated by Equations (21) and (23) are listed in Table 2. The shear stress at where sensor 4, sensor 5, and sensor 7 located calculated by Equations (22) and (24) are listed in Table 3, respectively.

### 3.3. Shear Stress Measured by LDV System 

**Figure 10:**
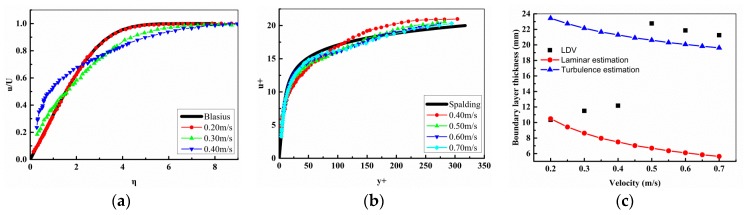
(**a**) Velocity profiles obtained by Laser Doppler Velocimetry (LDV) compares with laminar Blasius velocity profile. (**b**) Velocity profiles obtained by LDV compares with turbulent Spalding velocity profile. (**c**) Boundary layer thickness calculated by velocity profiles compared with laminar and turbulence empirical formulas values.

Meanwhile, the velocity profile at where sensor 5 located (x = 804 mm) were obtained by the DANTEC Flow Explorer LDV system, its uncertainty of velocity measurement is about 0.06%. An adequate near-wall resolution in the measurements is essential for an accurate interpretation of the results [25]. The velocity profiles compares with laminar Blasius [26] and turbulent Spalding [27]; velocity profiles are shown in Figure 10a,b, respectively. Hence, the shear stress can be obtained according to Equation (1). On the basis of boundary layer thickness definition [1], we can also get the boundary layer thickness according to the velocity profiles. The boundary layer thickness and shear stress that were calculated by the velocity profiles are shown in Table 4.

The boundary layer thickness derived from the velocity profiles compared with the values calculated by laminar and turbulence empirical formulas are shown in Figure 10c. From Figure 10 we can conclude that when the free-stream velocity is less than 0.2 m/s, the flow at x = 804 mm is laminar. When the velocity is great than 0.5 m/s, the flow is turbulence. And the flow is laminar to turbulence transition when velocity is greater than 0.2 m/s and less than 0.5 m/s.

**Figure 11:**
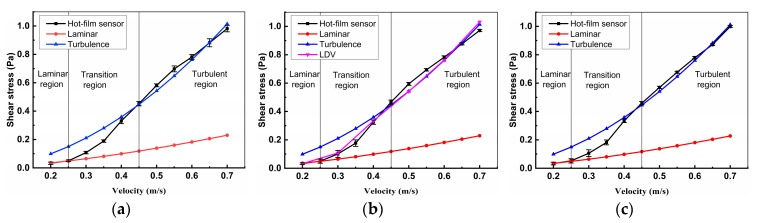
Shear stress measured by the hot-film sensors compared with the empirical formula and velocity profile. (**a**) Sensor 4; (**b**) Sensor 5; and, (**c**) Sensor 7.

The shear stress and its error [28] measured by the hot-film sensors compared with empirical formulas and LDV results were shown in Figure 11. We can see that the shear stress measured by the hot-film sensors are almost equal to the laminar empirical formulas values when the flow is laminar and basically same with the turbulence empirical formulas values when the flow is turbulence. From Figure 11b, we can see that the shear stress measured by sensor 5 (x = 804 mm) are all same with the LDV results no matter in laminar, transition, and turbulent regions. The measuring errors of the hot-film sensors are less than 5%.

## 4. Transition Measurement

In addition to quantitative shear stress measurement, the hot-film sensors are also developed to meet the requirements for boundary layer transition detection. The transition experiment facility, flat plate model, and data acquisition system are primarily the same as that described in the last chapter and Figure 9, but the sensor array was replaced by another one with eight functional sensors. The first sensor of the array (sensor 1) is 780 mm (x = 780 mm) from the leading edge of the flat plat, and the distance between two adjacent sensors is 6mm. Sensor 1 was used as a temperature probe to monitor the water temperature, and the other seven sensors were utilized to detect boundary layer transition.

There are three transition Reynolds numbers of interest to be resolved from the hot-film sensor signals [29]. These Reynolds numbers are the onset of transition, the 50% intermittent, and the end of transition. Characterized by the hot-film sensor output voltages, the beginning of transition is a low amplitude, steady-state signal with very occasional higher amplitude, high frequency bursts. The RMS value of the voltage at this Reynolds number is slightly higher than the RMS value of the lower Reynolds number. The 50% intermittent of the transition is characterized by large amplitude fluctuations, the RMS value of the signal is greatest. The hot-film signal at the end of transition is very random and moderate. The RMS value of this signal is higher than the laminar, but lower than the 50% intermittent transition.

**Figure 12:**
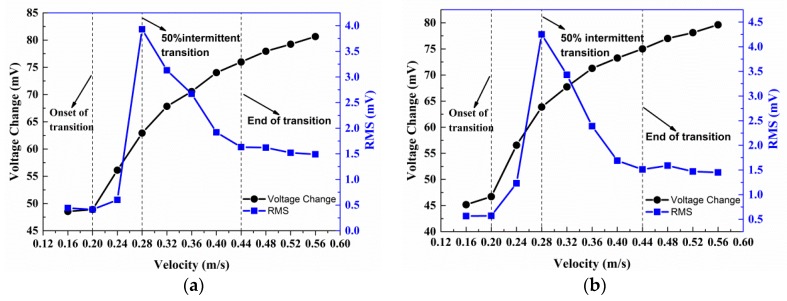
Voltage changes and RMS values of (**a**) sensor 2 and (**b**) sensor 8.

Two typical examples of the data for natural transition obtained from sensor 2 (x = 786 mm) and sensor 8 (x = 822 mm) are shown in Figure 12a,b, respectively. The RMS values of the fluctuating voltages have been normalized by the mean voltages as an indication of local turbulence level at the respective sensor locations. As seen in Figure 12, the mean voltage changes of sensor 2 and sensor 8 both have an abrupt increase at U = 0.2 m/s (Re = 1.82 × 10^5^). Meanwhile, the normalized RMS of sensor 2 and sensor 8 have a sudden increase at U = 0.2 m/s and reach a local maximum value at U = 0.28 m/s (Re = 2.52 × 10^5^), and then the RMS dropped sharply until U = 0.44 m/s (Re = 3.96 × 10^5^). When velocity is greater than 0.44 m/s, the RMS have very little or no change.

**Figure 13:**
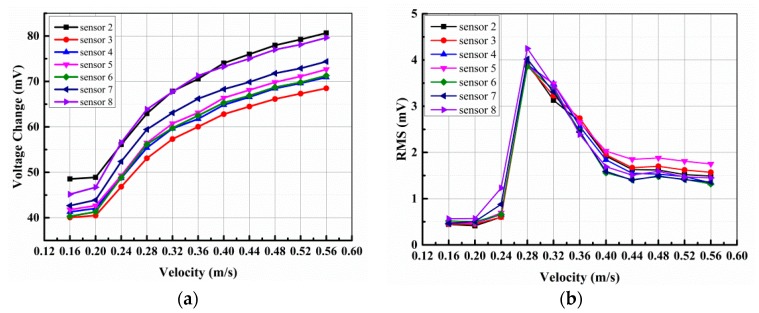
(**a**) Voltage change and (**b**) RMS value of all seven sensors.

The voltage changes and RMS of all seven sensors in the array are shown in Figure 13a,b, respectively. The variation tendency of other five sensors are same with sensor 2 and sensor 8. The voltage changes and RMS both have an abrupt increase at U = 0.2 m/s. The RMS reach a local maximum value at U = 0.28 m/s, then dropped sharply until U = 0.44 m/s. When velocity is greater than 0.44 m/s, the RMS reach a stable value which is greater than laminar flow and less than 50% intermittent transition.

In conclusion, at the region where the sensor array located on the flat plate, the transition begins at Re = 1.82 × 10^5^, 50% intermittent transition happens at Re = 2.52 × 10^5^ and the transition terminates at Re = 3.96 × 10^5^.

**Figure 14:**
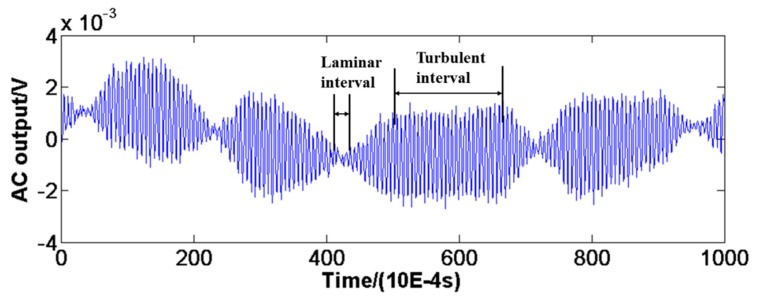
An AC output voltage-versus-time trace of sensor 2 at U = 0.28 m/s.

An AC output voltage-versus-time trace (Figure 14) of sensor 2 at U = 0.28 m/s clearly shows the movement of the transition region with time. Although this was actually the 50% intermittent transition when visually averaged, it is clear that the trace over the 0.1 s period shows both a laminar and turbulent interval.

**Figure 15:**
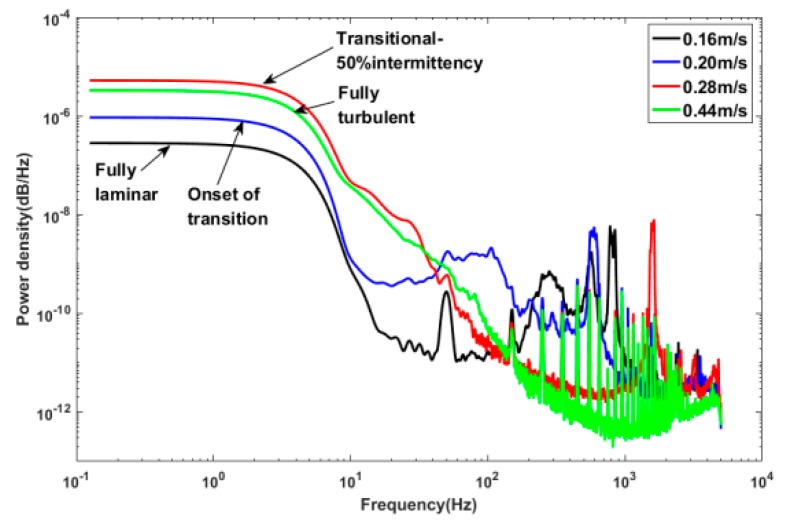
A waterfall plot of the spectra from the voltage-time traces of sensor 2 at different velocities.

A waterfall plot of the spectra (Figure 15) from the voltage-time traces of sensor 2 at different velocities indicate the characteristic shapes of laminar, transitional, and fully turbulent boundary layers. Welch’s power spectral density estimate method was employed to analyze the spectra of the output voltage signals, window is 2048 and noverlap is 1024. The source for the peak in energy at about 50 Hz that is present at U = 0.16 m/s is believed to be associated with power frequency interference, which is easy to be seen in laminar boundary layer spectra. The spectra of the low velocity (U = 0.16 m/s) shows a significantly low energy level, which is usually associated with a laminar boundary layer. The spectra at U = 0.20 m/s indicates the beginning of the transition and shows a marked increase in energy when compared to the lower velocities. The spectra at U = 0.28 m/s shows a surge in energy across the spectrum, which is associated with the 50% intermittency transitional boundary layer. The spectra for the fully turbulent boundary layer (U = 0.44 m/s) have a litter lower energy level than the 50% intermittency signal, but the energy is higher than the fully laminar and the onset of transition.

## 5. Conclusions

A flexible hot-film sensor array with Parylene C waterproof layer has been developed for quantitative underwater shear stress measurements. The calibrated hot-film sensors are able to measure the underwater shear stress accurately. Compared with the shear stress values measured by the LDV and calculated by empirical formulas, the measuring errors of the hot-film sensors are less than 5%.

The sensors are also able to detect boundary layer transition, including onset, 50% intermittency and end of transition by the use of mean voltage change, normalized RMS, and power spectra. The signals of the sensors indicate that the transition on the flat plat begins at Re = 1.82 × 10^5^, 50% intermittency transition occurs at Re = 2.52 × 10^5^, and ends at Re = 3.96 × 10^5^.

## Figures and Tables

**Table 1:** Shear stress measured by hot-film sensors.

Velocity (m/s)	Shear Stress (Pa)		
	Sensor 4 (x = 798 mm)	Sensor 5 (x = 804 mm)	Sensor 7 (x = 816 mm)
0.20	0.033	0.033	0.033
0.25	0.050	0.050	0.052
0.30	0.108	0.101	0.106
0.35	0.189	0.177	0.182
0.40	0.331	0.325	0.334
0.45	0.453	0.466	0.459
0.50	0.582	0.595	0.570
0.55	0.698	0.694	0.677
0.60	0.782	0.783	0.780
0.65	0.882	0.877	0.871
0.70	0.982	0.971	0.999

**Table 2:** Boundary layer thickness calculated by empirical formulas (x = 804mm).

Velocity (m/s)	Boundary Layer Thickness (mm)	
	Laminar Empirical Formula	Turbulence Empirical Formula
0.20	10.49	23.43
0.25	9.43	22.73
0.30	8.63	22.15
0.35	7.97	21.66
0.40	7.50	21.29
0.45	7.03	20.90
0.50	6.70	20.61
0.55	6.36	20.30
0.60	6.10	20.07
0.65	5.85	19.83
0.70	5.64	19.62

**Table 3:** Shear stress calculated by empirical formulas.

Velocity (m/s)	Shear Stress (Pa)					
	Laminar Estimation			Turbulence Estimation		
	x = 798	x = 804	x = 816	x = 798	x = 804	x = 816 (mm)
0.20	0.035	0.035	0.035	0.099	0.099	0.099
0.25	0.049	0.049	0.049	0.150	0.150	0.149
0.30	0.065	0.064	0.064	0.210	0.210	0.210
0.35	0.081	0.081	0.080	0.280	0.280	0.279
0.40	0.099	0.099	0.098	0.359	0.359	0.358
0.45	0.119	0.118	0.117	0.447	0.446	0.445
0.50	0.139	0.138	0.137	0.543	0.543	0.542
0.55	0.160	0.160	0.158	0.649	0.648	0.646
0.60	0.183	0.182	0.181	0.762	0.761	0.760
0.65	0.206	0.205	0.204	0.884	0.883	0.882
0.70	0.230	0.229	0.228	1.015	1.014	1.012

**Table 4:** Boundary layer thickness and shear stress measured by LDV.

Velocity (m/s)	Boundary Layer Thickness (mm)	Shear Stress (Pa)
0.20	10.35	0.033
0.30	11.50	0.108
0.40	12.17	0.334
0.50	22.76	0.542
0.60	21.86	0.763
0.70	21.25	1.031
